# PD-1 Regulates Neural Damage in Oligodendroglia-Induced Inflammation

**DOI:** 10.1371/journal.pone.0004405

**Published:** 2009-02-06

**Authors:** Antje Kroner, Nicholas Schwab, Chi Wang Ip, Christoph Leder, Klaus-Armin Nave, Mathias Mäurer, Heinz Wiendl, Rudolf Martini

**Affiliations:** 1 Department of Neurology, University of Wuerzburg, Wuerzburg, Germany; 2 Section of Developmental Neurobiology, University of Wuerzburg, Wuerzburg, Germany; 3 Clinical Research Group for Multiple Sclerosis and Neuroimmunology, University of Wuerzburg, Wuerzburg, Germany; 4 Department of Neurogenetics, Max-Planck-Institute of Experimental Medicine, Goettingen, Germany; Julius-Maximilians-Universität Würzburg, Germany

## Abstract

We investigated the impact of immune regulatory mechanisms involved in the modulation of the recently presented, CD8+ lymphocyte mediated immune response in a mouse model of oligodendropathy-induced inflammation (PLPtg-mutants). The focus was on the role of the co-inhibitory molecule PD-1, a CD28-related receptor expressed on activated T- and B-lymphocytes associated with immune homeostasis and autoimmunity. PLPtg/PD-1-deficient double mutants and the corresponding bone marrow chimeras were generated and analysed using immunohistochemistry, light- and electron microscopy, with particular emphasis on immune-cell number and neural damage. In addition, the immune cells in both the CNS and the peripheral immune system were investigated by IFN-gamma elispot assays and spectratype analysis. We found that mice with combined pathology exhibited significantly increased numbers of CD4+ and CD8+ T-lymphocytes in the CNS. Lack of PD-1 substantially aggravated the pathological phenotype of the PLPtg mutants compared to genuine PLPtg mutants, whereas the PD-1 deletion alone did not cause alterations in the CNS. CNS T-lymphocytes in PLPtg/PD-1-/- double mutants exhibited massive clonal expansions. Furthermore, PD-1 deficiency was associated with a significantly higher propensity of CNS but not peripheral CD8+ T-cells to secrete proinflammatory cytokines. PD-1 could be identified as a crucial player of tissue homeostasis and immune-mediated damage in a model of oligodendropathy-induced inflammation. Alterations of this regulatory pathway lead to overt neuroinflammation of high pathogenetic impact. Our finding may have implications for understanding the mechanisms leading to the high clinical variability of polygenic or even monogenic disorders of the nervous system.

## Introduction

We have recently investigated a mouse myelin mutant overexpressing the proteolipid protein (PLP) in oligodendrocytes leading to myelin degeneration and late onset axonal degeneration. Although being primarily caused by the glial mutation, the neuropathological phenotype was accompanied by an elevation of CD11b+ macrophages and CD8+ T-lymphocytes in the central nervous system [1]. Reconstitution experiments with RAG-1 deficient myelin mutants, receiving bone-marrow from either CD8+/CD4− or CD8−/CD4+ mutants, clearly identified CD8+ T-lymphocytes cells as pathogenic mediators. Lack of the macrophage-restricted molecule sialoadhesin in the PLP mutants, that mediates interactions of macrophage-like cells and T-lymphocytes [2], abrogates the elevation of CD8+ T-lymphocytes and substantially ameliorates the myelin-phenotype of the PLP mutants, further supporting the pathogenetic role of CD8+ cells in PLP transgenic mice [3]. In this model, CD8+ lymphocytes show clonal expansions in the diseased CNS but not in peripheral lymphatic organs. This serves as a strong hint for a pathogenetic, antigen-specific role of these cells [4]. The link between oligodendrocyte damage and components of the adaptive immune system is particularly relevant for inflammatory disorders of the nervous system. It has been recently hypothesized that subtypes of multiple sclerosis (MS) may be caused by a primary oligodendropathy [5], [6]. This hypothesis is strongly supported by recent clinical reports, showing that PLP mutations in humans can be linked to primary progressive or relapsing-remitting MS [7], [8]. Thus, our recent work identifying a primary oligodendropathy as a “trigger” for immune-driven pathological changes is important for our understanding of pathomechanisms occurring in some forms of MS.

It is well known, that genetic and environmental factors control disease onset and disease course of CNS inflammatory autoimmune disorders [9]. The co-inhibitory molecule “programmed death” (PD)-1 (CD279) is a CD28-related receptor expressed on activated T- and B-lymphocytes and associated with immune homeostasis and autoimmunity [10], [11]. Accordingly, we recently demonstrated that a polymorphism of the PD-1 gene is associated with a progressive disease course in MS [12], therefore corroborating the importance of PD-1 as a disease modifying gene. Moreover, inactivation of PD-1 in an animal model for inherited demyelination in the peripheral nervous system implicating T-lymphocytes [13] leads to a substantial aggravation of the primarily genetically-caused neuropathy [14].

In the present study, we investigate the impact of immune-regulatory mechanisms involved in the modulation of the immune response in PLP transgenic mice, a model of oligodendropathy-induced inflammation. Our present study identifies PD-1 as a crucial factor regulating tissue homeostasis of T-lymphocytes and indicates that a primary oligodendropathy combined with alterations in this regulatory pathway can lead to accelerated neuroinflammatory reactions of high pathogenetic impact.

## Materials and Methods

### Animals and Determination of Genotypes

PLP transgenic mice [15] were bred and genotyped as described previously [1]. PD-1-/- mice [16] were kindly provided by T. Honjo and C. Blank. Absence of PD-1 was verified by PCR genotyping as described [14]. To generate double mutants, PLPtg and PD-1-/- mice were crossbred. All resulting genotypes were investigated at 2, 6 and 12 months of age.

To exclude organ autoimmunity in the PD-1-/- mice [16], urine samples were investigated for protein and glucose with CombiScreen® urine tests (BioconDiagnostik, Voehl-Marienhagen, Germany). For bone marrow chimerization, PLPtg/RAG-1-/- mice were used as recipients. RAG-1 deficiency was identified as previously described [1], [14], [17].

All mice were bred and kept in our animal facility under barrier conditions (University of Wuerzburg, Department of Neurology). All animal experiments were approved by the local authorities (Regierung von Unterfranken).

### Bone marrow chimerization

Two strategies to generate bone marrow chimeras were performed: In one group, the recipients (PLPtg mice with PLPwt mice as controls) were sublethally irradiated (5 Gy), while mice in the other group were deficient for the recombination activating gene (RAG)-1 (PLPtg/RAG-1-/- recipients with PLPwt/RAG-1-/- as controls). The bone marrow chimeras were transplanted with PD-1-/- or wildtype bone marrow at the age of 6–8 weeks and investigated at the age of 10 months (n = 8–9). Transplantation and control of successful transplantation was performed as described before [1], [18], [19].

### Purification of splenocytes

Spleens were passed through a cell strainer (BD Biosciences Pharmingen, San Jose, CA USA), erythrocytes were lysed with a lysis buffer (150mM NH_4_Cl_2,_ 10 mM KHCO_3_, 0.1 mM EDTA in distilled water at pH 7.3) and cells were washed and processed for the respective experiments.

### Preparation of CNS mononuclear cells and flow cytometry of splenocytes and CNS lymphocytes

Mice were killed with CO_2_ and transcardially perfused with cold 0.1 M PBS. The CNS was prepared, tissues were homogenized and cells were gradient isolated as described [1]. Flow cytometry was performed using standard methods as described [4], [20].

### Tissue preparation and immunohistochemistry

For identification of macrophage-like cells, mice were transcardially perfused with 4% paraformaldehyde in 0.1 M cacodylate buffer. Tissue was dissected, postfixed for 2 hours and cryoprotected in 30% sucrose overnight. For T- lymphocyte and MBP staining, mice were perfused with 0.1 M phosphate buffered saline (PBS) only. After snap freezing, 10 µm thick transverse sections of the spinal cord and longitudinal or transverse sections of the optic nerve were cut.

Immunohistochemistry for CD11b, Sialoadhesin, CD4, CD8 and MBP was performed as described before [1], [3]. MBP was stained on optic nerve cross sections 1200–1400 µm caudal to the retina. Sources of reagents, of antibodies and clones of the antibodies were the same as described [1], [3].

### Assessment of demyelination

Myelin damage in optic nerve cross sections was assessed by measuring MBP negative areas, data were displayed as a percentage of the total area. For this, we used a Zeiss Axiophot2 microscope at a final magnification of 300×. The area was measured using digital images acquired via a CCD- camera and ImagePro 4.0 software.

Additionally, myelin damage was semiquantitatively rated as described before [1], with score 1 depicting homogeneous MBP distribution, score 5 massive myelin loss.

### Tissue preservation for light microscopy of semithin sections

Optic nerves from transcardially perfused mice were processed for light microscopy of semithin sections as recently reported [1]. Tissue damage was assessed by quantification of axonopathic vacuoles >6 µm.

### Quantification of immune cells in the CNS

Longitudinal sections of the optic nerve of 2, 6 and 12 months old wildtype, PD-1-/-, PLPtg and PLPtg/PD-1-/- mice and 10 months old bone marrow chimeras were analysed. Quantification of CD11b+ and Sialoadhesin (Sn)+ cells was performed as described before [1] in the rostral region. CD4+ and CD8+ T-cells were quantified in total longitudinal optic nerve sections, using a Zeiss Axiophot2 microscope and measurement tools as described above.

### Detection of cytokines by ELISA and ELISPOT

1×10^6^/ml splenocytes were cultured unstimulated or stimulated with ConA (2 µg/ml, Sigma, Schnelldorf, Germany) or CD3/CD28 coated microspheres (Dynal, Invitrogen, Karlsruhe, Germany). After 48 hours, supernatant was harvested and ELISA for IFN-γ, IL-2 or IL-10 (R&D Systems, Minneapolis, MN, USA) was performed according to the manufactureŕs instructions.

Assessment of interferon-gamma (IFN-γ) producing cells was performed by ELISPOT. 1x10^4^ CNS lymphocytes or 1×10^5^ splenocytes per well were incubated for 24 hours, unstimulated or stimulated with PMA (20 ng/ml)/Ionomycin (500 ng/ml, both Sigma), or a mixture of class one PLP, MOG and MBP peptides (Genscript Corp, Piscataway, NJ, USA) as previously described [4]. ELISPOT assay was performed according to the manufactureŕs instructions (BD Pharmingen). Spots were quantified by CTL Europe (Aalen, Germany) using ImmunoSpot 4.0.17.

### CDR3 Spectratyping

The CDR3 spectratyping was performed as described previously [4], [21] using an ABI Prism 3130 capillary sequencer (Applied Biosystems) to determine length and distribution, using a module for fragment-analysis. As an internal length standard, 500-ROX (Applied Biosystems) was used.

### Statistical analysis

Quantified profiles were tested with two-tailed student's t-test, scores were analyzed by the nonparametric Mann-Whitney-U test and Kruskal-Wallis test.

## Results

The role of PD-1, a co-inhibitory molecule critical for immune homeostasis and tolerance, was tested in a model of CNS-myelinopathy associated with secondary, low grade inflammation of high pathological relevance.

### Numbers of CNS immune cells are significantly elevated in PLPtg/PD-1-/- double mutants

To obtain myelin mutants with inactivated PD-1 function, two strategies have been chosen. First, double mutants have been created in analogy to previous experiments [1], [3] by crossbreeding the corresponding single mutants. This strategy has the advantage that all cells of the organism lack PD-1. Complementarily, bone marrow chimeric mutants have been generated using either irradiated or RAG-1-deficient PLPtg mice as recipients and PD-1-/- mice as donors [1], [18]. The latter strategy has the advantage that unexpected side effects or influences of the systemic PD-1-inactivation (e.g. influences of PD-1 deficiency on thymic maturation or neonatal tolerance) can be circumvented.

PLPtg/PD-1-/- double mutants and their respective controls (wt, PD-1-/- and PLPtg mice), were examined at the age of 2, 6 and 12 months (n = 3–7). To quantify immune cells in the CNS, we focussed on longitudinal sections of the optic nerve, an already established read out technique for the scoring of inflammation in PLP mutant CNS [1], [3], [22].

**Figure 1:**
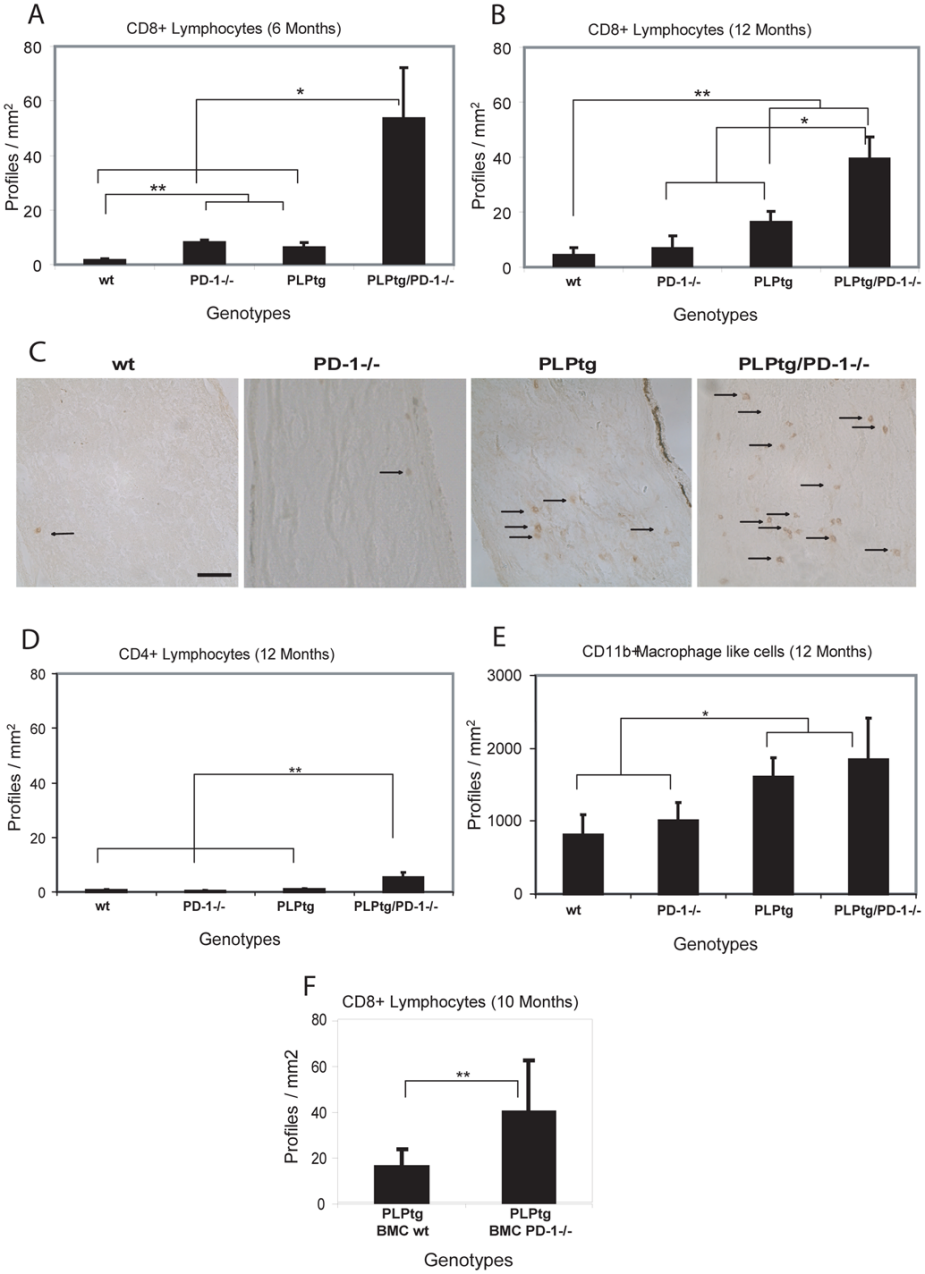
Quantification and immunohistochemical detection of CD8+ and CD4+ lymphocytes and CD11b+ macrophage-like cells in longitudinal sections of optic nerves of wt, PD-1-/-, PLPtg and PLPtg/PD-1-/- mice and of CD8+ cells in bone marrow chimeric mice - A, B. Quantification of CD8+ lymphocytes in 6 (A: n = 3) and 12 (B: n = 3–7) months old mice of different genotypes. C. Immunohistochemical detection of CD8+ lymphocytes in the optic nerve of 12 months old mice of different myelin and immunological genotypes. Arrows indicate positively labelled CD8+ lymphocytes. D. Quantification of CD4+ lymphocytes in 12 months old mice (n = 3–7). Note that in the myelin mutants, both CD8+ and the generally scarce CD4+ lymphocytes are substantially increased in the absence of PD-1. E. Quantification of CD11b+ macrophage-like cells in 12 months old mice (n = 3–6). Note that the number CD11b+ cells does not differ significantly in PLPtg/PD-1+/+ and PLPtg/PD-1-/- double mutants. Similar results are obtained in irradiated and non-irradiated (RAG-1-/-) PLPtg bone-marrow recipients. The rather small increase of CD11b+ cells is not surprising, since the molecule in focus (PD-1) is a component of predominantly T-lymphocytes rather than of macrophages/microglial cells. F. Quantification of CD8+ cells in the CNS of 10 months old PLPtg bone marrow chimeras (BMCs) which were transplanted with either wt or PD-1-/- bone marrow (n = 8–9). Note that also in bone marrow chimeras, CD8+ T-lymphocytes are substantially elevated in the CNS of PLPtg mutants in the absence of PD-1. Error bars represent standard deviations. * p- value<0.05, ** p - value≤0.01. Scale Bar: 50 µm.

At the age of two months, there was no significant difference between wt, PD-1-/-, PLPtg and PLPtg/PD-1-/- mice. At 6 months, however, there was a slight elevation of CD8+ T- cells in the optic nerve of PLPtg mice in comparison to wildtype mice, consistent with our previous observations. PD-1-/- also showed a mild elevation, whereas PLPtg/PD-1-/- double mutant mice exhibited a robust upregulation of CD8+ T-cells in optic nerve sections (approximately 35-fold increase compared to wildtype mice and a more than 8 fold increase in comparison to PLPtg mice, Figure 1A). In 12 months old mice, the general pattern of CD8+ lymphocyte numbers in optic nerves was similar in the different genotypes, but proportions had shifted a little: wt and PD-1-/- mice were now at similar levels (with no marked increase of profiles in PD-1-/- mice compared to the 6 months old group), and PLPtg mice displayed significantly more CD8+ profiles than the former two genotypes. Strikingly, these CD8+ T-lymphocytes were again clearly outnumbered by those from PLPtg/PD-1-/- mice (Figure 1B, C).

CD4+ T-lymphocytes are rarely present in the CNS of PLPtg and wt mice [1]. At the age of two months, no differences were detectable in all investigated groups.

At the age of 6 months a significant elevation of CD4+ lymphocytes was already visible in PLPtg/PD-1-/- compared to wt, PD-1-/- and PLPtg mice and there was no marked increase until the age of 12 months (Figure 1D). Similar changes in T-lymphocyte numbers were detected in the spinal cord (data not shown).

Furthermore, we investigated the number of CD11b+ macrophage-like cells in the optic nerve. We did not detect significant differences at the age of 2 or 6 months, although a trend of increased numbers was already detectable in PLPtg mice and PLPtg/PD-1-/- double mutants at 6 months (data not shown). In 12 months old mice, however, a difference was detectable. While wt and PD-1-/- mice showed a common low level of cells, both PLPtg and PLPtg/PD-1-/- mice had elevated numbers of CD11b+ cells (Figure 1E).

We additionally investigated the number of Sialoadhesin (Sn) expressing macrophage like cells in 12 months old PLPtg/PD-1-/- mice and detected a very low amount of positive profiles in both wildtype and PD-1-/- mice. In PLPtg, a more than ten-fold increase was detectable, but there was no significant difference between PLPtg and PLPtg/PD-1-/- mice (data not shown).

### Numbers of immune cells are significantly elevated in PLPtg PD-1-/- transplanted bone marrow chimeras (BMCs)

Another strategy to investigate the role of PD-1 in PLPtg mice was to examine PLPtg mice which were transplanted with bone marrow from either PD-1-/- mice (PLPtg BMC PD-1-/-) or wildtype mice (PLPtg BMC wt). Wildtype mice which were transplanted with either wildtype or PD-1-/- bone marrow served as controls. These animals never displayed myelin pathology or CNS inflammation in any experiment.

The bone marrow chimeric mice faithfully reflected the findings described for the double mutants. For example, both irradiated and PLPtg/RAG-1-/- recipients showed a robust upregulation of CD8+ lymphocytes, when bone marrow was derived from PD-1-/- mice (Figure 1F). Similar observations were made for the low but significantly elevated amount of CD4+ cells (data not shown). Increased elevation of CD8+ and CD4+ cells in the absence of PD-1 was significant both in irradiated and in RAG-1-deficient PLPtg mice.

Analysis of CD11b+ macrophage like cells depicted a small, significant increase in PLPtg PD-1-/- transplanted chimeras compared to recipients that received bone marrow from wildtype mice (data not shown). In the irradiated PLPtg mice statistical significance was reached, while the PLPtg/RAG-1-/- mice showed a trend into the same direction but did not reach the level of significance (p = 0.06).

### Pathological features are enhanced in PLPtg/PD-1-/- double mutated mice

**Figure 2:**
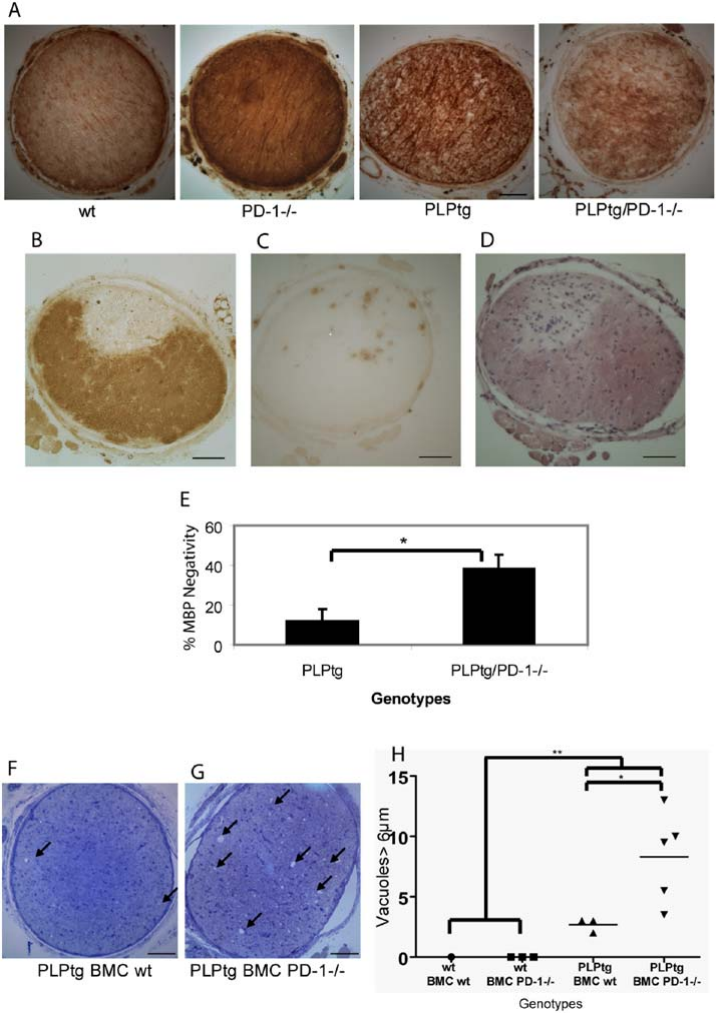
Quantification of pathological features in various myelin and PD-1 mutants and BMC mice A. MBP immunohistochemistry of optic nerve cross sections of 12 months old wt, PD-1-/-, PLPtg and PLPtg/PD-1-/- mice. Note homogeneous MBP distribution in wt and PD-1-/- mice compared to more disrupted myelin (inhomogeneous labeling) in PLPtg and, more pronounced, PLPtg/PD-1-/- mice. B.-D. MBP immunohistochemistry (B), immunohistochemical detection of CD8+ cells (C) and hematoxylin eosin staining (D) in a plaque like demyelinating lesion in a 12 months old PLPtg/PD-1-/- mouse. Note sharply confined lesion (B) with accumulated T-cells (C) and other, probably inflammatory, cell nuclei (D). E. Quantification of demyelination by measuring MBP-negative areas. PD-1-deficient myelin mutants (PLPtg/PD-1-/-) show more severe MBP loss than PLPtg mice expressing PD-1 (PLPtg; n = 4–6). F.,G. Semithin optic nerve sections sections of 10 months old PLPtg mice which were transplanted with wt (F) or PD-1-/- (G) bone marrow. Arrows indicate periaxonal vacuoles which are more numerous in PD-1-/- BMCs. H. Quantification of vacuoles >6 µm in bone marrow chimeras. PD-1-deficiency leads to the most robust histopathological alterations in the myelin mutants and the most seriously affected mutants belong to the PD-1-deficient group (n = 3–5). * p- value<0.05, ** p - value≤0.01. Scale bars: 50 µm.

MBP immunohistochemistry revealed that, while wildtype and PD-1-/- mice always showed a homogeneous distribution of myelin, PLPtg mice displayed a more patchy and inhomogeneous MBP staining. Compared to that, PLPtg/PD-1-/- mice showed an even less homogeneous MBP distribution, reflecting extensive myelin loss (Figure 2A).

Interestingly, in one PLPtg/PD-1-/- optic nerve we observed an extended and sharply confined area of MBP-loss, reminiscent of a demyelinated lesion common to active or inactive MS plaques (Figure 2B). This lesion was associated with an accumulation of CD8+ lymphocytes (Figure 2C) and hematoxylin-stained cells of probably inflammatory character (Figure 2D).

To quantify the demyelinating phenotype of the different mutants, MBP-negative areas of optic nerve cross sections were determined as measure for demyelination. Neither wildtype nor PD-1-/- mice showed any MBP-negative areas, while demyelination was present in PLPtg mice and further significantly increased in PLPtg/PD-1-/- mice (Figure 2E). Additionally, myelin integrity-related MBP distribution was analysed by semi-quantitative scoring. Again, wt and PD-1-/- mice showed healthy myelin (score 1) while PLPtg mice had an average score of 3±0.81 and PLPtg/PD-1-/- mice showed a higher score (3.83±0.75). PD-1-deficiency leads to the highest MBP loss in the myelin mutants and the most seriously affected mutants belong to the PD-1-deficient group. Generally, the persons investigating the histopathological features (A. K., R. M.) were not aware of the respective genotypes. Similar to these results in double mutant mice, 10 months old PLPtg BMCs reconstituted with PD-1-/- bone marrow showed a more disrupted state of MBP distribution than PLPtg mice transplanted with BMCs from wt mice (data not shown).

We also investigated periaxonal vacuoles in semithin cross sections of the optic nerve as another reliable pathological marker [1], [3] (age of 12 months in double mutants, age of 10 months in bone marrow chimeras). PLPtg/PD-1-/- double mutants, compared to PLPtg mice, showed a clear trend of increased vacuole numbers (13.3±7.9 versus 7.95±3.6), while wt and PD-1-/- mice never displayed any vacuoles (data not shown).

PLPtg BMCs transplanted with PD-1-/- bone marrow showed a highly significant increase of axonal damage (Figure 2F, G, H). In the optic nerves of wildtype mice, we never detected any vacuoles, regardless what kind of bone marrow had been transplanted.

### T-cell CDR3 spectratype analysis: robust clonal expansions in the CNS of PLPtg/PD-1-/- mice

Evidence for monoclonal T-cell expansions as revealed by single Vβ-Jβ peaks in the corresponding PCR-diagrams could be detected (one Vβ peak per animal) in 12-month-old PLPtg mice corroborating these previous findings [4]. Corresponding expansions could not be detected in spectratyping analyses from lymphocytes of wild type mice. Spleens of the same animals displayed the expected Gaussian distribution of Vβ profiles [4].

**Figure 3:**
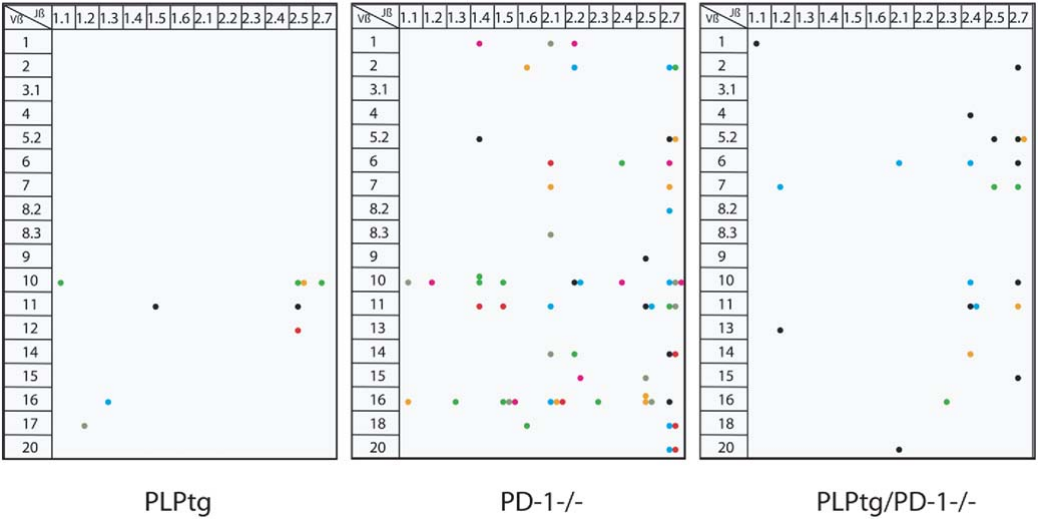
Spectratyping of CNS derived lymphocytes - Lymphocytes from the CNS of 12 months old PLPtg (n = 7, as adopted from [4], PD-1-/- (n = 7), and PLPtg/PD-1-/- (n = 4) mice were analysed for disturbances in the T-cell receptor repertoire by spectratyping. Clonal expansions (visible as single peaks in the fragment analysis) are shown as colored dots (different colors indicate individual animals). The expanded T-cells are characterized by their Vβ- and Jβ-chains. The clonal expansions occurred widely distributed over different Vβ and Jβ regions, although some domains seemed to be prone for clonal expansions in different mutant mice. Note that different numbers of experimental mice contribute to the different numbers of dots.

CDR3 spectratype in PD-1-/- mice (n = 7) showed more than one Vβ peak (Figure 3). Similarly, multiple Vβ peaks were visible in the CNS of PLPtg/PD-1-/- double mutants (n = 3) (Figure 3), as well as in 10 months old PLPtg/PD-1-/- BMCs (n = 5). The clonal expansions occurred widely distributed over different Vβ and Jβ regions, although some domains seemed to be prone for clonal expansions in different mutant mice. Notably, approximately 30% of the individual clonal expansions were detected in both spleen and brain, 70% of the expansions were exclusively present in CNS tissue.

We then sequenced some clones to 1) demonstrate that the specific PCR fragment represents one TCR (a readable sequence proves the existence of an expansion), and 2) to detect similarities between the CDR3s of different clonal expansions. For example, by analysing the sequences of 2 individuals (mouse A and B) we identified two expansions with the same VβJβ combination and one expansion with a different VβJβ combination in the two mice (Table S1). While those D segments with flanking N sequences ( = NDN) amino acids, responsible for connecting with the MHC-bound antigen, show some similarities, the lengths of the CDR3 are not identical, showing that these clones are not specific for the exact same antigen.

### Peripheral immune parameters do not differ between mutant mouse strains

In order to exclude that peripheral immune parameters in the mutant mouse strains could account for the different numbers of immune cells and the aggravated pathological features in double mutated mice, we analyzed (i) phagocytic capability of macrophages, (ii) inducibility and rate of stimulation-induced apoptosis of splenocytes, (iii) immune subset distribution (CD4+, CD8+, CD11b+, B220), and (iv) levels of stimulation induced IL-2 production between the different groups (wt, PD-1-/-, PLPtg, PLPtg/PD-1-/-).

The phagocytic capacity of peritoneal macrophages was similar in all genotypes. Furthermore, investigation of splenocytes showed no significant differences between the different genotypes used in this study in regard of inducibility of apoptosis, cell subsets and production of cytokines (see Material S1, Figure S1).

### CNS T-cells are prone to IFN-γ secretion in the absence of PD-1

Polyclonal immune responses in the periphery do not differ between mouse mutants. We therefore tested whether CNS cells show altered production of inflammatory cytokines upon stimulation. IFN-γ secretion of CNS-derived T-lymphocytes was measured after addition of PMA/ionomycin or upon antigenic stimulation.

**Figure 4:**
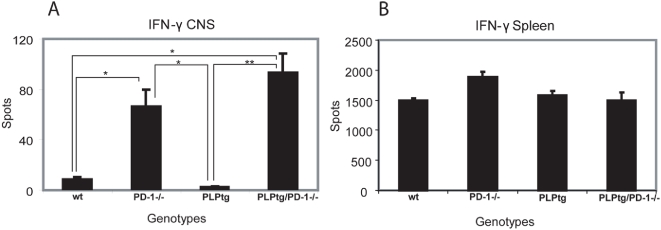
IFN-γ ELISPOT assay on spleen and brain derived lymphocytes - IFN-γ ELISPOT assay after stimulation with PMA/ionomycin on CNS derived lymphocytes (A) and splenocytes (B) from wt, PD-1-/-, PLPtg and PLPtg/PD-1-/- mice. Note elevated spot numbers in CNS T-lymphocytes taken from PLPtg/PD-1-/- mice in comparison to wt and PLPtg mice, reflecting a higher susceptibility to activation. Error bars represent standard deviations. * p - value<0.05, ** p - value≤0.01

Interestingly, CNS lymphocytes of PD-1-/- and PLPtg/PD-1-/- mice showed strong IFN-γ secretion upon PMA/ionomycin challenge, while CNS T-cells from PLPtg mice or wt showed only minimal or no cytokine production (Figure 4A). Of note, such differences have not been observed in T-cells from spleen (Figure 4B). Antigenic stimulation with a number of MHC class I related myelin peptides [4] did not lead to IFN-γ production of CNS T-cells under any condition (ELISPOT, data not shown).

## Discussion

Recent data from human studies in distinct leukodystrophies and some forms of MS together with investigations in myelin mutant mice indicate that a primary glial injury can be causative for neuroinflammation of substantial pathological and clinical relevance [1], [3], [4], [23], [24], [25]. Further characterization of “secondary” inflammatory responses in a model of PLP overexpression identified CD8+ T-lymphocytes of effector cell phenotype as crucial mediators of demyelination and axon damage [1]. The finding that CD8+ T-cells in the CNS of myelin mutants are clonally expanded [4], [25] further supports a pathogenetic concept that primary myelin damage in the CNS can be associated with secondary reactivity of the adaptive immune system against a still unknown antigen(s). Another key question in this scenario is which factors control tissue homeostasis of immune cells. We therefore investigated the impact of immune-regulatory mechanisms on the adaptive immunity in PLP overexpressing mutants focussing on the role of the co-inhibitory molecule PD-1. Recent data in mice as well as in humans demonstrate that PD-1 is substantially involved in the control of T-cell homeostasis under physiological and pathological conditions [26] by preventing uncontrolled proliferation of autoreactive T-cells [27]. In accordance with these functional data, certain polymorphisms in the PD-1 gene are associated with human autoimmune disease including MS [12], [28].

Our present experiments show that PD-1 plays a major role in modulating numbers of CD8+ cells in the demyelinating model of PLP overexpressing mice.

The most relevant finding of our study was that in PLPtg mice the histopathological phenotype was much more severe when PD-1 was absent. Interestingly, not only CNS damage was more pronounced per individual, but most severely affected individuals always belonged to the group of PLPtg mice in combination to functional disruption of PD-1.

How does ablation of the PD-1 pathway affect CNS pathology? PD-1-deficiency alone only transiently affected the number of CD8+ cells found in the CNS and, more importantly, the corresponding mice showed normal histological features in the CNS. While it has been reported that PD-1-/- mice on a C57/Bl6 background show lupus-like glomerulonephritis when aging [16] we did not observe any obvious organ pathology, urinal glucose or protein content (not shown) or spontaneous autoreactions associated with loss of PD-1. Furthermore, the peripheral immune “status” was not changed in the tested groups (wt, PD-1-/-, PLPtg, PLPtg/PD-1-/-). However, we found clonal expansions of T-cells in the periphery in PD-1-/- mice using CDR3 spectratyping analysis. While numerically not elevated in the CNS at 12 months, the PD-1-deficient CD8+ cells of PLPwt/PD-1-/- mice showed multiple clonal T-cell expansions, as opposed to PD-1-expressing CD8+ cells of normal wild type mice which do not show any repertoire perturbations. The corresponding CD8+ cells of PLPtg mice display their characteristic mono- or oligoclonal expansion in the CNS as previously described [4]. The combination of the PLP transgene with PD-1 deficiency led to significantly higher numbers of T-cells within the CNS. Moreover, CD8+ T-lymphocytes of PD-1 mutants are aberrant with regard of their strong numerical increase and prominent clonal expansions. This suggests that PD-1 prevents a large number of possible clonal expansions of a variety of T-cell clones in PLPtg mice. PD-1 signalling is known to attenuate signals of the T-cell receptor such as PKCtheta and ZAP-70/CD3ζ [29]. This could also be the explanation that only CNS T-cells deficient of PD-1 show markedly enhanced INF-γ secretion after stimulation, whereas CNS CD8+ cells from genuine PLPtg mice show no relevant production of this proinflammatory cytokine. These findings suggest that although prominently expanded and highly susceptible to become activated, PD-1-deficient T-lymphocytes appear to be pathologically “silent” in a healthy environment.

Due to its inhibitory properties the cognate ligand PD-L1 has been proposed to contribute to maintaining peripheral tolerance and limiting inflammatory damage [20], [27], [30], [31], [32]. Parenchymal PD-L1 contributes to the limitation of insulinitis and the resolution of inflammation [33]. We and others recently reported that PD-L1 is expressed and upregulated on CNS cells (e.g. microglia cells) under inflammatory conditions [20], [34], restricts parenchymal neuroantigen-specific T-cell responses and confines inflammatory CNS damage in experimental autoimmune encephalomyelitis [35]. One therefore might assume that PD-L1 - PD-1 interactions counteract T-cell mediated pathology observed in PLPtg mice by limiting clonal expansion and cytokine release of detrimentally self-reactive low avidity clones.

A synoptic view summarizing our recent and previous observations may be as follows: overexpression of PLP may induce intracellular stress that causes several immune-relevant glial reactions, such as expression of cytokines and upregulation of MHC-I molecules on oligodendrocytes [1] and Sn on the surface of macrophage-like cells. It is of note that the latter reaction is an important prerequisite for CD8+ cell activation in the present model [3]. Furthermore, supraphysiological concentrations of myelin antigens associated with PLP-overexpression could promote reactivity of low-avidity T-cell clones that survived clonal deletion or ignorance in the thymus [4]. CNS-derived, but not spleen-derived CD8+ cells show mono- or oligoclonal expansions, further suggesting CNS-restricted specificity against yet unidentified CNS-antigens. PD-1 is critically involved in these processes: in the presence of PD-1 on CNS CD8+ cells, activation and proliferation is limited, whereas absence of PD-1 leads to substantial increase of CD8+ cells, a higher propensity to secrete proinflammatory cytokines, multiple clonal expansions and an aggravation of neural damage.

Taken together, our study demonstrates the important role of a co-inhibitory molecule, PD-1, in modulating glial-injury-related immune responses in the CNS. This impressively reflects the high and obviously wide-range relevance of immunmodulatory mechanisms under various pathological conditions and should be particularly considered when seeking for mechanisms leading to the high clinical variability of polygenic or even monogenic disorders of the nervous system.

## Supporting Information

Figure S1 Exclusion of differences in the peripheral immune systems of wt, PD-1-/-, PLPtg and PLPtg/PD-1-/- double mutants. Peritoneal macrophages were incubated with fluorescent latex beads and the percentage of macrophages which ingested beads was found similar in all genotypes (A). Differences in the apoptosis rate were excluded by flow cytometry of annexin V and PI positive splenocytes under highly stimulatory conditions (B). Analysis of immune cell subsets (CD4+, CD8+, CD11b+ and B220+ cells) showed similar distribution in splenocytes of all investigated genotypes (C). Exclusion of deviations in peripheral proinflammatory activation using unstimulated and stimulated splenocytes from different genotypes, by examining IL-2 in the corresponding supernatants by ELISA (D). Error bars represent standard deviations.
(0.39 MB TIF)

Material S1 Material and Methods S1
(0.03 MB DOC)

Table S1 CDR3 sequences. Sequencing analysis of two clones with identical VβJβ combinations from PLPtg/PD-1-/- mice A and B and of one clone with a different VβJβ combination from mouse A. Note identical TCRVβ and TCRJβ regions surrounding the CDR3 region, which not only differs in aminoacids but also, more importantly, in length, thus indicating recognition of different antigens.
(0.03 MB DOC)
