# Correction: PD-1 Regulates Neural Damage in Oligodendroglia-Induced Inflammation

**DOI:** 10.1371/annotation/51612e49-7bbe-4a84-b88d-fc5b7d79d853

**Published:** 2011-01-11

**Authors:** Antje Kroner, Nicholas Schwab, Chi Wang Ip, Christoph Leder, Klaus-Armin Nave, Mathias Mäurer, Heinz Wiendl, Rudolf Martini

The authors wish to add the following to the Competing Interests Section: "Dr. Wiendl has received funding for travel and speaker honoraria from Bayer Schering Pharma, Biogen Idec/Elan Corporation, Sanofi-Aventis, Merck Serono, and Teva Pharmaceutical Industries Ltd.; has served/serves as a consultant for Merck Serono, Medac, Inc., Sanofi-Aventis/Teva Pharmaceutical Industries Ltd., Biogen Idec, Bayer Schering Pharma, Novartis, and Novo Nordisk; and receives research support from Bayer Schering Pharma, Biogen Idec/Elan Corporation, Sanofi-Aventis, Merck Serono, and Novo Nordisk."

