# Supplementary figures and images for: PD-1 Regulates Neural Damage in Oligodendroglia-Induced Inflammation
Source: PLoS One. 2009 Feb 6;4(2):e4405. doi: 10.1371/journal.pone.0004405 (PMC2635015; doi:10.1371/journal.pone.0004405)

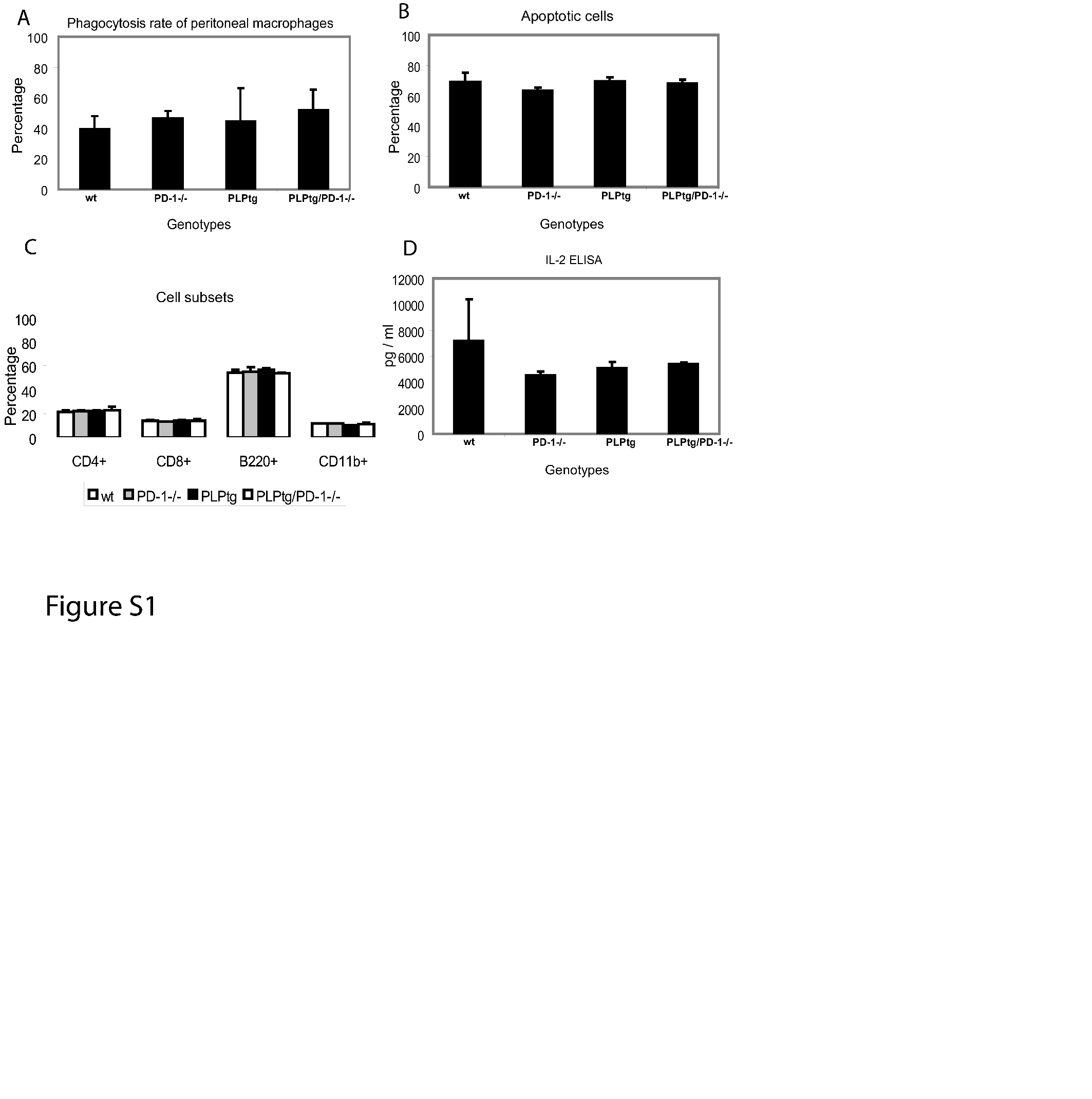

Supplement: Figure S1 — Exclusion of differences in the peripheral immune systems of wt, PD-1-/-, PLPtg and PLPtg/PD-1-/- double mutants. Peritoneal macrophages were incubated with fluorescent latex beads and the percentage of macrophages which ingested beads was found similar in all genotypes (A). Differences in the apoptosis rate were excluded by flow cytometry of annexin V and PI positive splenocytes under highly stimulatory conditions (B). Analysis of immune cell subsets (CD4+, CD8+, CD11b+ and B220+ cells) showed similar distribution in splenocytes of all investigated genotypes (C). Exclusion of deviations in peripheral proinflammatory activation using unstimulated and stimulated splenocytes from different genotypes, by examining IL-2 in the corresponding supernatants by ELISA (D). Error bars represent standard deviations. (0.39 MB TIF) [file pone.0004405.s001.tif]
