# Supplementary material for: PD-1 Regulates Neural Damage in Oligodendroglia-Induced Inflammation
Source: PLoS One. 2009 Feb 6;4(2):e4405. doi: 10.1371/journal.pone.0004405 (PMC2635015; doi:10.1371/journal.pone.0004405)
Supplement: Material S1 — Material and Methods S1 (0.03 MB DOC) [file pone.0004405.s002.doc]

**Material and Methods S1**

**Phagocytosis rate of peritoneal macrophages**

Mice were killed by CO_2_, peritoneal macrophages were obtained by injection of 5 ml of ice cold PBS into the peritoneal cavity and the fluid containing peritoneal macrophages was extracted. The peritoneal fluid was centrifuged, cells were counted and taken into culture (Medium: RPMI with 10 % FCS , 1 % Penicillin and Streptomycin, 1 % Glutamate (all from Gibco, Invitrogen, Karlsruhe, Germany)) in a concentration of 1x 10^6^ cells/ml and incubated for 24 hours at 37°C in a humified atmosphere with 5 % CO_2_/ 95 % air.

For examination of phagocytosis rate, fluorescent polystyrene latex beads (Fluoresbrite® YG Microspheres 2.00µm, Polysciences Inc., Warrington, PA, USA) were first incubated with 1% BSA for 30 minutes, then medium was added and the mixture was added to macrophages in a tenfold concentration and incubated for 30 minutes in the dark.

Afterwards, cells were washed five times with ice cold PBS, then detached using EDTA trypsin (Gibco, Invitrogen, Karlsruhe, Germany) for five minutes. Cells were washed once more in FACS buffer (0.1 M PBS containing 0.1% BSA and 0.1 % sodiumazide) and analysed by flow cytometry. Positive labelling was visualized by fluorescence intensity using flow cytometry analysis, ingestion of more than one fluorescent bead resulted in proportional increase of fluorescence intensity.

**Induction and quantification of apoptosis**

3 x 10^6^ Splenocytes were cultured in splenocyte complete medium (10 mM HEPES, 25 µg/ml gentamicin, 50 µM mercaptoethanol, 5 % FCS, 2 mM glutamine, 1 % NEAA, all from Sigma, Schnelldorf, Germany) with maximal stimulation (Concanavalin A (ConA) 5 μg/ml (Sigma, Schnelldorf, Germany) or 3 x 10^6^ CD3/CD28 microspheres (Dynal, Invitrogen, Karlsruhe, Germany) for 48 hours. Afterwards, cells were washed in the permeabilization buffer (1 mM HEPES, 150 mM NaCl, 5 mM KCl, 1 mM MgCl_2_, 0.18 mM CaCl_2_, all from Sigma, Schnelldorf, Germany) and stained with FITC labelled Annexin V (Roche, Mannheim, Germany) and 50 µg/ml Propidium Iodide (Calbiochem, Merck, Nottingham, UK). Analysis was performed by flow cytometry.
