# Supplementary material for: PD-1 Regulates Neural Damage in Oligodendroglia-Induced Inflammation
Source: PLoS One. 2009 Feb 6;4(2):e4405. doi: 10.1371/journal.pone.0004405 (PMC2635015; doi:10.1371/journal.pone.0004405)
Supplement: Table S1 — CDR3 sequences. Sequencing analysis of two clones with identical VβJβ combinations from PLPtg/PD-1-/- mice A and B and of one clone with a different VβJβ combination from mouse A. Note identical TCRVβ and TCRJβ regions surrounding the CDR3 region, which not only differs in aminoacids but also, more importantly, in length, thus indicating recognition of different antigens. (0.03 MB DOC) [file pone.0004405.s003.doc]

| PLPtg/PD1-/-  mouse | TCRVβ  region | TCRVβ  sequence | NDN  region | TCRJβ  sequence | TCRJβ  region |
| --- | --- | --- | --- | --- | --- |
| A | 5.2 | CASSL | EGGGEQY | FGPGTRL | 2.7 |
| B | 5.2 | CASSL | QGEY | FGPGTRL | 2.7 |
| A | 11 | CASSL | GDSQNTLY | FGAGTRL | 2.4 |
